# Editorial: Exploring human subjectivity

**DOI:** 10.3389/fpsyg.2023.1146775

**Published:** 2023-02-13

**Authors:** Esteban Laso Ortiz, Luis Angel Saúl, Luis Botella

**Affiliations:** ^1^Centro Universitario de la Ciénega, Universidad de Guadalajara, Guadalajara, Mexico; ^2^Facultad de Psicología, Universidad Nacional de Educación a Distancia (UNED), Madrid, Spain; ^3^Facultat de Psicologia, Ciències de l'Educació i l'Esport Blanquerna, Universitat Ramon Llull, Barcelona, Spain

**Keywords:** subjectivity, linguistics, computing, artificial intelligence, machine learning, natural language processing, communality, neuropsychology

In their eagerness to systematize knowledge, the social sciences have come up against a *prima facie* insurmountable obstacle throughout their history: subjectivity. From Wittgenstein's problem of private language (Wittgenstein, 1953; Kripke, 2004) to the impossibility of interpersonal comparisons of utility (Lemieux, 2022), to the controversy over the measurement of happiness (Gardiner et al., 2020), or the debate over the very possibility of measuring psychological phenomena (Michell, 1999), the specter of subjectivity is always present when it comes to transcending the gulf between one individuality and another.

It can be said, therefore, that (inter)subjectivity is the frontier of the social sciences, which makes it an apt subject for this issue of Frontiers.

The texts in this issue reflect the breadth and scope of the problem of subjectivity in science, in its empirical, methodological and theoretical aspects. Beginning with the latter, the theoretical article of the issue addresses subjectivity from one of its facets, the study of consciousness. In recent decades the materialist assumptions underlying positivist scientific practice have been sharply questioned for their inability to explain consciousness (the “hard problem,” Melloni et al., 2021) without appealing to ontological dualism. From these questionings has emerged the alternative of the ontological primacy of consciousness; that is, that the ultimate foundation of reality is consciousness (and, hence, subjectivity), from which matter is derived (Bitbol, 2008). This position frames the article “*Is Consciousness First in Virtual Reality?*”, which takes virtual reality (VR) as a paradigm for conceiving reality as a product of consciousness via perception and explores the requirements and implications of this assumption (Slater and Sanchez-Vives).

Subjectivity is present at the methodological level as the challenge of individualizing and measuring “objectively” (i.e., independent of perception) the subjective aspects of the human condition, from personality traits to experiences or meanings. The authors of “*Rigorous idiography: Exploring subjective and idiographic data with rigorous methods—The method of derangements*” relate this contrast between subjectivity and objectivity to two others common in the psychological literature: between the idiographic (information about a single individual) and the nomothetic (information that allows the individual to be compared with peers on a given dimension), and between the qualitative and the quantitative (Evans et al.). The aim of his article is to bridge the gap between these poles by proposing an elegant but statistically rigorous method for testing whether purely subjective, idiographic and qualitative data contain reliable and objective information: the method of derangements.

Another way to bridge this gap has been the use of mixed techniques, among which the Repertory Grid technique (Osterberg-Kaufmann, 2022) stands out. The brief report “*Self-concept 6 months after traumatic brain injury and its relationship with emotional functioning”* shows that this technique is able to demonstrate changes in the self-concept of patients who have suffered traumatic brain injuries, correlating them with life satisfaction, anxiety and quality of life (Mascialino et al.).

The influence of subjectivity on behavior is analyzed in “*Going green is exhausting for dark personalities but beneficial for the light ones: An experience sampling study that examines the subjectivity of pro-environmental behavior*,” an article that makes use of ecological momentary assessment, which allows for the collection of informants' experiences as they emerge naturally from the context (Kesenheimer and Greitemeyer). The authors show that people with pro-environmental attitudes and/or “light” personality (faith in humanity, humanism and kantism) not only behave in a pro-environmental way in daily life but also perceive pro-environmental behaviors as less burdensome and more advantageous than people with “dark” personality (Machiavellianism, sadism, narcissism or psychopathy).

The last text of the monograph goes into the field of subjectivity par excellence: psychotherapy. The aim of “*Identification of dynamic patterns of personal positions in a patient diagnosed with borderline personality disorder and the therapist during change episodes of the psychotherapy*” is to study the processes of subjective transformation of a patient diagnosed with borderline personality disorder (Mellado et al.). To this end, the authors identify key episodes of change (defined as transformations in the patient's subjective theories of self) and, within these, the self-states that both the patient and the therapist go through (as judged by the dialogic positions they adopt *vis-à-vis* the interlocutor). In this way, they present evidence for the existence of dynamic interactive patterns between patient and therapist positions and their impact on the latter's improvement.

As can be seen, the five articles in this issue address the problem of subjectivity from different angles, in various fields and with different methodologies, so we are sure that they will be of interest to researchers who dare to tread the tempestuous sea of subjectivity in science.

## Author contributions

EO wrote the first draft of the manuscript. All authors contributed to manuscript revision, read, and approved the submitted version.

## References

[B1] Bitbol M. (2008). Is consciousness primary? NeuroQuantology 6, 53–72. 10.14704/nq.2008.6.1.157

[B2] Gardiner G. Lee D. Baranski E. Funder D. Members of the International Situations Project (2020). Happiness around the world: a combined etic-emic approach across 63 countries. PLoS ONE 15, e0242718. 10.1371/journal.pone.0242718 33296388 PMC7725360

[B3] Kripke S. A. (2004). Wittgenstein on Rules and Private Language: An Elementary Exposition (Reprinted. ed.). Oxford: Blackwell.

[B4] Lemieux P. (2022). Interpersonal Comparisons of Utility. Econlib. Available online at: https://www.econlib.org/interpersonal-comparisons-of-utility/ (accessed January 15, 2023).

[B5] Melloni L. Mudrik L. Pitts M. Koch C. (2021). Making the hard problem of consciousness easier. Science 372, 911–912. 10.1126/science.abj3259 34045342

[B6] Michell J. (1999). Measurement in Psychology: A Critical History of a Methodological Concept. Cambridge: Cambridge University Press. 10.1017/CBO9780511490040

[B7] Osterberg-Kaufmann N. (2022). Innovating empirical research on legitimacy: repertory grid analysis. Front. Polit. Sci. 4, 832250. 10.3389/fpos.2022.832250

[B8] Wittgenstein L. (1953). Philosophical Investigations: The German Text, with a Revised English Translation. Oxford: Blackwell.

